# Supplementary material for: UV-C Promotes the Accumulation of Flavane-3-ols in Juvenile Fruit of Grape through Positive Regulating VvMYBPA1
Source: Plants (Basel). 2023 Apr 18;12(8):1691. doi: 10.3390/plants12081691 (PMC10144632; doi:10.3390/plants12081691)
Supplement: Supplementary file 1 [file plants-12-01691-s001.zip › Supplementary files/plants-2306560-supplementary Figure1 and Table 1.docx]

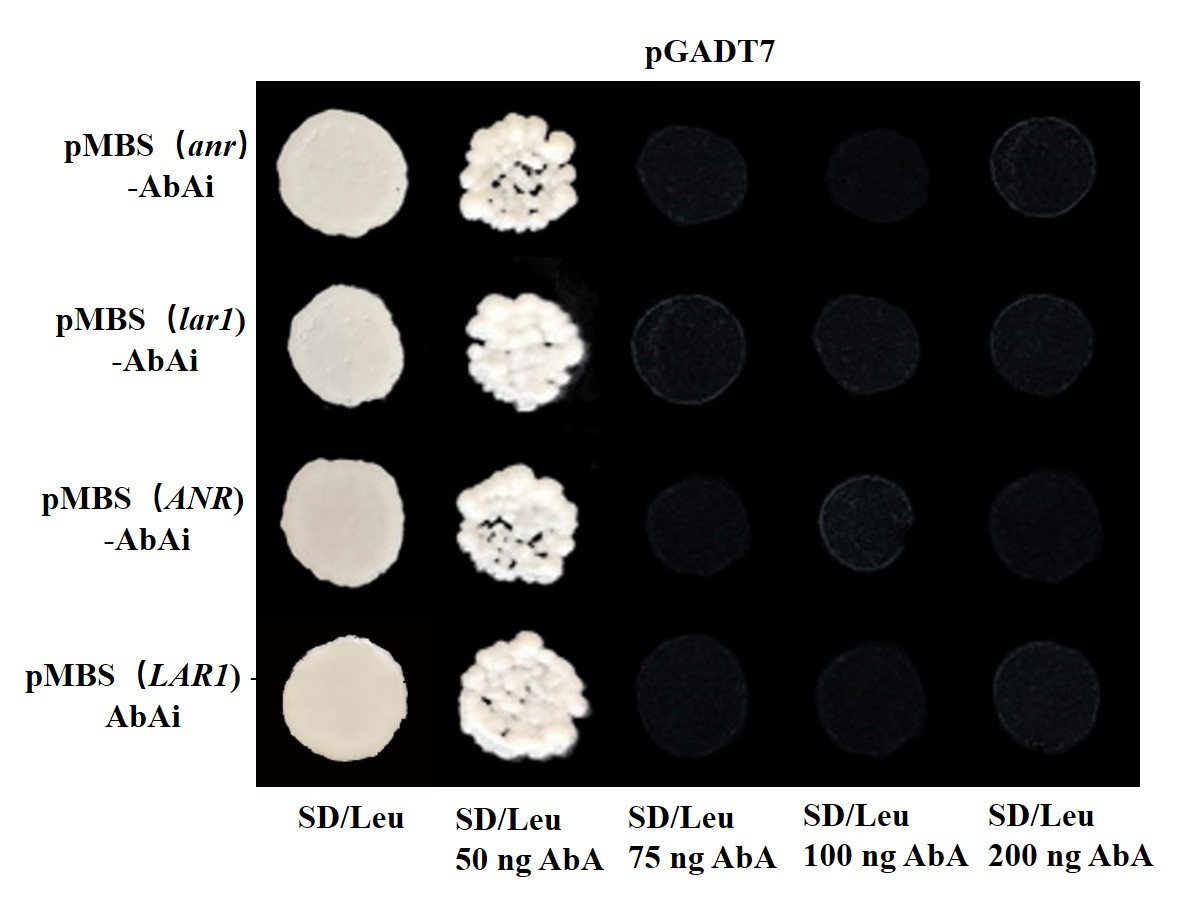


**Supplementary Figure 1. Concentration screening experiment of AbA in Yeast one hybrid.**

**Supplementary Table 1. All primers used in this study**

| Primers name | Primiers sequence | Product fragment length (bp) | Restriction enzyme |
| --- | --- | --- | --- |
| GFP-*VvMYBPA1*-F | CGGAGCTCATGGGCAGAGCACCTTGTT | 858 | *Sac*I |
| GFP-*VvMYBPA1*-R | GCGGATCCAATGAGTAGTGATTCGG |  | *BamH*I |
| OX-*VvMYBPA1*-F | CAAGCTTATGGGCAGAGCACCTT | 861 | *Hind* III |
| OX-*VvMYBPA1*-R | GGCGTCGACTTAAATGAGTAGTG |  | *Sal* I |
| RT-*VvActin*-F | CCCCATGCTATCCTTCG | 125 |  |
| RT-*VvActin*-R | AGGCAGCTCATAGTTC |  |  |
| RT-*VvANR*-F | AGAACTACAGGAGTTGGGTGAC | 202 |  |
| RT-*VvANR*-R | CCTTGAATTGCTGGCTTG |  |  |
| RT-*VvLAR1*-F | ACGATGTCCGAACACTGAAC | 187 |  |
| RT-*VvLAR1*-R | TGAACGCCGCTACTACACTC |  |  |
| RT-*VvLAR2*-F | TCTCGACATACATGATGATGTG | 166 |  |
| RT-*VvLAR2*-R | TGCAGTTTCTTTGATTGAGTTC |  |  |
| RT*-VvMYBPA1*-F | GTCTCTCATCGCAGGTAGGC | 190 |  |
| RT*-VvMYBPA1*-R | GCTGATCTTGACCCTCTTGC |  |  |
| BD-*VvWDR1*-F | CGGAATTCATGGAGAGATCAAGCC | 1011 | *Eco*R I |
| BD*-VvWDR1*-R | CGGGATCCCTAAACTTTAAGAAGC |  | *Bam*H I |
| BD*-VvMYC2-*F | GGAATTCCATATGATGAAAACTGAAATGGG | 1827 | *Nde* I |
| BD-*VvMYC2-*R | TCCCCCGGGTTACCCAACTGATGATGAC |  | *Sma* I |
| AD*-VvMYC2-*F | CGCCCGGGATGAAAACTGAAATGGGTATG | 1827 | *Sma* I |
| AD*-VvMYC2-*R | GGCGTCGACTTACCCAACTGATGATGAC |  | *Sal* I |
| BiFC-*MYBPA1*-F | CGAATTCATGGGCAGAGCACCTT | 858 | *Eco*R I |
| BiFC-*MYBPA1*-R | GGCGTCGACAATGAGTAGTGATTCGGCGAA |  | *Sal* I |
| BiFC-*VvWDR1*-F | CGGATCCATGGAGAGATCAAGCCT | 1008 | *Bam*H I |
| BiFC-*VvWDR1*-R | GGCGAATTCAACTTTAAGAAGCTGCAGTTT |  | *Eco*R I |
| BiFC-*VvMYC2*-F | GGCGTCGACATGAAAACTGAAATGGGTATG | 1824 | *Sal* I |
| BiFC-*VvMYC2*-R | GGACTAGTTTACCCAACTGATGATGAC |  | *Spe* I |
| Y1H-*VvANR*-F | CGAGCTCGTTAGTTGGGAACCATC | 204 | *Sac* I |
| Y1H-*VvANR*-R | GCGTCGACGCATATCTCAACAGCAG |  | *Sal* I |
| Y1H-*VvLAR1*-F | CGAGCTCACATAAATCCGGCCTAG | 119 | *Sac* I |
| Y1H-*VvLAR1*-R | GCGTCGACTGACTCACCATTCATGA |  | *Sal* I |
